# A new standard model for milk yield in dairy cows based on udder physiology at the milking-session level

**DOI:** 10.1038/s41598-017-09322-x

**Published:** 2017-08-21

**Authors:** Patrick Gasqui, Jean-Marie Trommenschlager

**Affiliations:** 1 Auvergne – Rhône-Alpes Center, Animal Health Division, National Institute for Agricultural Research, Animal Epidemiology Research Unit, EPIA, INRA, VetAgro Sup, 63122 Saint Genès Champanelle, France; 2 Nancy-Lorraine Center, Science for Action and Development Division, National Institute for Agricultural Research, Mirecourt Farm, Domaine du Joly BP 35, 88501 Mirecourt, France

## Abstract

Milk production in dairy cow udders is a complex and dynamic physiological process that has resisted explanatory modelling thus far. The current standard model, Wood’s model, is empirical in nature, represents yield in daily terms, and was published in 1967. Here, we have developed a dynamic and integrated explanatory model that describes milk yield at the scale of the milking session. Our approach allowed us to formally represent and mathematically relate biological features of known relevance while accounting for stochasticity and conditional elements in the form of explicit hypotheses, which could then be tested and validated using real-life data. Using an explanatory mathematical and biological model to explore a physiological process and pinpoint potential problems (i.e., “problem finding”), it is possible to filter out unimportant variables that can be ignored, retaining only those essential to generating the most realistic model possible. Such modelling efforts are multidisciplinary by necessity. It is also helpful downstream because model results can be compared with observed data, via parameter estimation using maximum likelihood and statistical testing using model residuals. The process in its entirety yields a coherent, robust, and thus repeatable, model.

## Introduction

In milk-yield studies, Wood’s model is the standard of reference^1–3^, in part because of its good fit to observed data, but mostly because no better alternatives exist^4–22^. A study in 1947^23^ first underscored the need for an explanatory model of milk production physiology to better understand and thus control udder mastitis^24, 25^, a problem that remains a major concern in the dairy industry; this need has yet to be met.

Historically, researchers have characterised milk production in cow udders by individually examining different system components, namely the dynamics of the alveolar population over the course of lactation^26–29^, of alveolar milk synthesis between milking sessions (accounting for different udder compartments and quarters)^30–34^, and of udder emptying during milking^35–37^. This work helped clarify the biological mechanisms at play. Subsequent research was able to go further in describing system components and demonstrating how they interacted in dairy cows. For example, scientists focused on the dynamics of alveolar populations and of milk synthesis^38–42^; the links between milk synthesis, udder compartments, and milking interval duration^43–52^; and the consequences of udder-emptying problems for milk yield^53–66^.

It is important to note that these biology-oriented models were based on daily milk yield rather than on session-specific milk yield. In 1991, Beever^67^ compared results obtained from empirical models with those from more mechanistic models, inspiring further research. Notably, Dijkstra^26^ (in 1997), Pollott^29, 68^ (in 2000 and 2004), and Hanigan^69^ (in 2007) developed daily-yield biological models in which parameter estimates could be adjusted to real-life observations. Other related work led to the creation and exploration of biological simulations of daily yield (e.g., Shorten^70^ in 2002). However, these models were overparameterised and thus parameter estimation from observed data was not possible.

Because the research described above was focused on daily milk yield and did not address udder-emptying dynamics or the precision of session-level measurements, it cannot be used to fully parse the variance associated with milk yield. Furthermore, such approaches cannot directly exploit data from automated milking systems, which are becoming more and more common. To obtain daily yields (i.e., for a 24-hour period) from such systems, additional calculations^71, 72^ must be used, which increases response variability.

Our goal was to exploit this previous research to construct a more holistic model of milk production. We therefore selected the information that was needed to properly represent per-session milk yield for a given animal over its entire lactation period. We used a maximum likelihood estimation approach and explicitly modelled the mean, variance, and covariance of the random variables that we studied. First, we present a simplified version of the model’s formalisation. We then provide and discuss the results. In the latter part of the methods, we address more technical details—the mathematics and statistics that were used to systematically develop the model.

### Model formalisation

**Figure 1:**
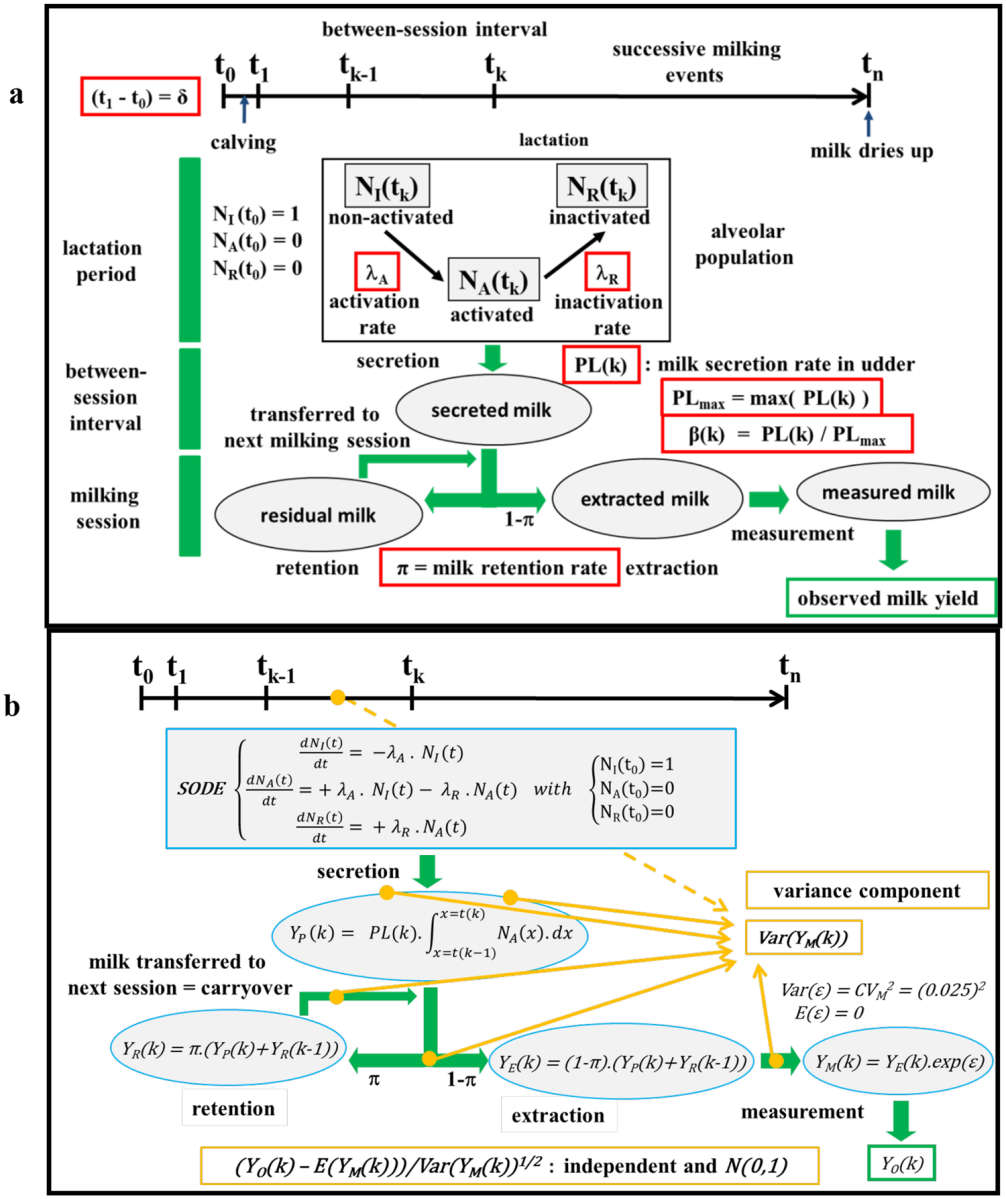
Model components. (**a**) Biological model and parameters and (**b**) mean and variance models.

Here, we present a mathematical representation of this biological process that has deterministic properties; it also has stochastic properties, which are framed by probabilities, management of randomness, and the potential influence of unobserved conditional elements (see Fig. 1 for model structure and Methods for the detailed model description). In our biological study system, cows are milked for the first time shortly after calving. Then, cows are milked twice a day, in the morning and the evening (mean interval durations: 14 hours between evening and morning milkings and 10 hours between morning and evening milkings; see Supplementary Fig. 1). For the total lactation period and each milking session, we know milk yield (a quantity whose precision is established *a priori*) for the four quarters of the udder; we also know the date and time of each milking session.

In our model, the udder comprises a single quarter with a single compartment. Over a given lactation period, the size of the alveolar population is constant; it is only renewed between lactation periods. Alveoli belong to one of three classes, whose proportions change over the lactation period^26, 29^. Time is expressed in hours since the beginning of lactation, which occurs prior to calving. Since the precise timing of this moment is unknown, it is necessary to estimate *δ*, which is the number of hours between the start of lactation and the first milking session. Once milk production has begun, changes in alveolar class proportions are described using a system of ordinary differential equations (SODE) with two parameters to estimate: alveolar activation rate, *λ*_*A*_, and alveolar inactivation rate, *λ*_*R*_, which are constant over the lactation period. Milk yield is modelled by considering that milk production between two successive milking sessions is a function of interval duration, the proportion of activated alveoli during the interval, and milk secretion rate^30, 32^. The latter remains constant between successive milking sessions. When modelling milk extraction during the milking session, it is assumed that the udder is not fully emptied^35–37^; the degree of extraction depends on the milk retention rate, π. Because the retention rate and the secretion rate are constant over the lactation period, we can use the normalized estimated residuals to calculate the following: 1) variation in the retention rate for a specific milking session *π*(*k*), which corresponds to the physiological fact that extreme retention rates are seen during certain milking sessions; and 2) variation among intervals in the milk secretion rate *PL*(*k*), which corresponds to the physiological fact that secretion rate changes over the lactation period. We could thus determine the interval-specific proportions *β*(*k*) = *PL*(*k*)*/max*(*PL*(*k*)), which are constant within intervals. From a methodological standpoint, these two variation types are conditional elements linked to variation-generating factors that act over the lactation period and that are not necessarily characterized *a priori*. In the variance model, the total variance for each milking session is broken down into individual components of stochasticity. Therefore, we could obtain a component’s proportional contribution for each milking session and for the entire lactation period. We also estimated the variance associated with interval duration, which can be treated as a random variable for which only mean and variance are known. A model describing daily production (hereafter, the daily-yield model) was derived from the results of the finer-scale, milking-session model. Once validated, its findings were compared with those obtained using Wood’s model.

## Results

The maximum likelihood estimation results (Tables 1 and 2 for milking-session model, and Table 3 for Wood’s model) are illustrated with data from the second lactation of a healthy Montbéliarde cow (i.e., never displayed symptoms of clinical mastitis).

**Table 1:** Estimation of the expected parameters of the milking-session model.

Exp. model parameter (unit)	Delta time *δ* (hour)	Activation rate *λ*_*A*_ (per hour)	Inactivation rate *λ*_*R*_ (per hour)	Retention rate min. *min*(*π*(*k*)) (proportion)	Secretion rate max. *max*(*PL*(*k*)) (kg per hour)
Estimate [95% CI]	70.98 [55.33; 91.05]	64.10^−4^ [55; 75].10^−4^	45.10^−6^ [38; 53].10^−6^	0.041 [0.022; 0.068]	1.41 [1.38; 1.43]

**Table 2:** Estimation of the variance parameters of the milking-session model.

Var. model parameter	Inflation variance factor *ϕ* (proportion)
Estimate [95% CI]	0.54 [0.43; 0.68]

**Table 3:** Estimation of the expected parameters for Wood’s model.

Parameter (Wood)	a	b	c
Estimate [95% CI]	19.392 [19.344; 19.439]	0.1777 [0.1769; 0.1785]	0.00376 [0.00375; 0.00377]

**Figure 2:**
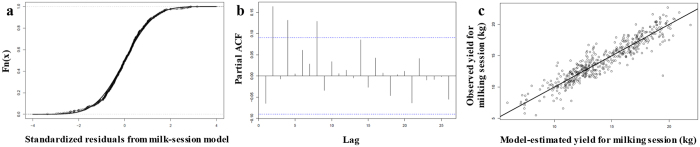
Validating the milking-session model. (**a**) Cumulative distribution of the model’s standardized residuals overlain by the normal cumulative distribution curve (KS test: P-value = 0.45). (**b**) Partial estimate of autocorrelation for the standardized residuals (Box-Pierce test: P-value = 0.16). (**c**) Plot of model-estimated versus observed yield for milking sessions (best-fit line in black; r = 0.93).

Following model validation (Fig. 2), we obtained normally distributed standardized residuals for the milking-session model and the daily-yield model (Kolmogorov-Smirnov [KS] test: P-value = 0.4458 and P-value = 0.7131, respectively). The residuals were also uncorrelated (Box-Pierce test: P-value = 0.1599 and P-value = 0.1169, respectively). For Wood’s model, the residuals were normally distributed (KS test: P-value = 0.5817) but auto-correlated (P-value < 10^−6^); consequently, the confidence intervals and the statistical tests using estimated variances were unreliable.

**Figure 3:**
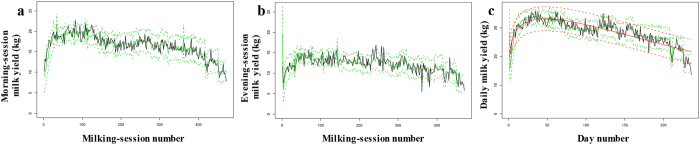
Observed versus estimated milk yield. Values for (**a**) morning milking sessions, (**b**) evening milking sessions, and (**c)**, full days. Observed yield is represented by a solid black line. Model-estimated yield and the 95% CI are represented by green lines (solid and dashed, respectively). The estimated yield from Wood’s model and the 95% CI are represented by red lines (solid and dashed, respectively).

The milking-session and daily-yield models appear to fit the observed data nicely (r = 0.93 and r = 0.92, respectively). For Wood’s model, the correlation was slightly weaker (r = 0.87). We then plotted the observed values alongside the estimated values obtained from three milking-session model types (i.e., morning milking, evening milking, and daily yield; ±95% CI). We estimated daily yield by aggregating the values for a day’s successive milking sessions; we then compared the results with those obtained using Wood’s model (Fig. 3).

**Figure 4:**
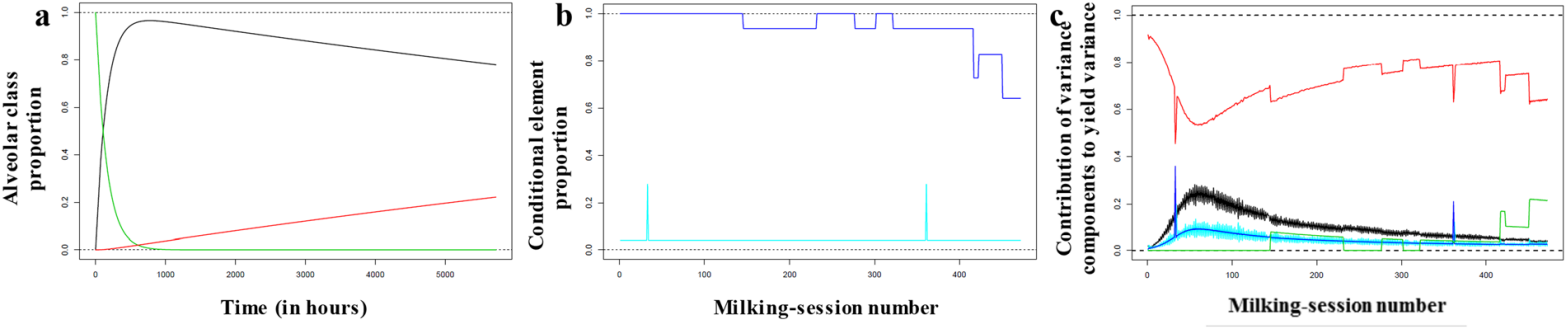
Estimates of unobserved variables. (**a)** Alveolar class proportions over time: non-activated alveoli (green line), activated alveoli (black line), and inactivated alveoli (red line). (**b**) Conditional elements operating during milking sessions: β (dark blue line) and π (light blue line). (**c**) Relative contribution of variance components to milking-session variance in yield (% provided is the estimate for whole lactation period): alveolar class proportions (78.4%; red line); measurement error (9.4%; black line); β (4.3%; green line); π (4.0%; blue line); and milk carryover (3.9%; cyan line).

Using the milking-session model, we estimated changes in the mean proportions of the alveolar classes over the lactation period. We also estimated changes in *β* (Table 4) and π (Table 5) over successive milking sessions. The estimated proportion of total variance explained by the milking-session model and the daily-yield model was 1.03 and 1.07, respectively. The variance model did a good job of accounting for total observed variance. Upon breaking down total variance, we found that variance in the proportion of activated alveoli made the largest contribution (total variance for the lactation period: 78.4%; total variance per session: Fig. 4).

**Table 4:** Estimation of the conditional elements *β* in the milking-session model.

Proportion β(k) estimate	1.00	0.94	0.83	0.73	0.64
Secretion rate PL(k) estimate (kg per hour) [95% CI]	1.41 [1.38; 1.43]	1.32 [1.28; 1.35]	1.16 [1.10; 1.23]	1.02 [0.92; 1.13]	0.90 [0.84; 0.96]

**Table 5:** Estimation of the conditional elements *π* in the milking-session model.

Milking number	33 and 361	others
Retention rate π(k) estimate [95% CI]	0.28 [0.19; 0.38]	0.04 [0.02; 0.07]

When precise interval duration was unknown, the proportion of total variance explained by the milking-session model and the daily-yield model was 0.98 and 1.07, respectively; total variance was higher than previously. As before, the proportion of activated alveoli made the largest contribution to total variance overall (62.1%) and nearly always to session-specific variance. However, the variance due to the limited information on interval duration was the second largest contributor (18.8%; see Supplementary Fig. 4). We estimated also daily yield by aggregating the values for a day’s successive milking sessions; we then compared the results with those obtained using Wood’s model (see Supplementary Fig. 5). When precise interval duration was unknown, the daily-yield model, like Wood’s model, was subject to “smoothing”. In such cases, our model could obtain estimates of characteristic lactation traits, such as the length of peak lactation (28 days for our model vs. 47 days for Wood’s model) and peak milk yield (33.8 kg for our model vs. 33.2 kg for Wood’s model)^73–75^. Total milk production per lactation period was 6,749 kg in the observed data, 6,744 kg in our model, and 6,750 kg in Wood’s model.

A simulation was used to examine the influence of the model’s four main parameters—*λ*_*A*_, *λ*_*R*_, *PL*_*max*_, and *π*—on the mean and variance models, as well as on the main model validation criteria (see Supplementary Fig. 6).

## Discussion

In summary, our biological model incorporates different sources of variability and provides a good fit for observed milking-session and daily yields. It uses data that are easy for livestock farmers to obtain, notably milk yields for all four quarters of the udder for each milking session and the length of time between successive sessions.

The proportion of activated alveoli over time is the largest contributor to variance; this variance component includes a dispersion parameter that accounts for underdispersion because of the interdependence of the alveolar classes. To characterise quarter-specific variability, quarter-specific milk yield per milking session must be quantified. Part of this variability could also stem from inconsistency in alveolar activation and inactivation rates across the lactation period or from the rates being quarter specific. However, our assumption of rate consistency was validated *ex post* because the model’s predictions agreed with the observed data.

Since interval duration is the main factor explaining milk yield, it needs to be described more precisely. When only mean duration is available, variance associated with this variable becomes the second greatest contributor to variance in milk yield. Consequently, it is clear that daily-yield models (where interval duration is a constant 24 hours) will provide less precise yield estimates. Moreover, such models cannot account for milk retention, meaning that the autocorrelation among residuals will render any statistical results unreliable; this is the case for yields obtained with Wood’s model. Our model could be used with automated milking systems (i.e., robotic systems) in which milking occurs every 8 hours on average, making it possible to model quarter-specific yields.

Our model is based on a small number of parameters that all have clear biological interpretations. It also accounts for the action of conditional elements via the use of two types of variation. Retention rate could explain the persistence of pathogenic agents in the udder and their subsequent migration into the alveolar compartment when the alveolus relaxes at the end of the milking session. Variation in *β*, which accounts for the influence of several potential conditional elements during the lactation period, can be used to test their effects *a posteriori*. It should be noted that interpreting *β* may be tricky because it is an umbrella variable that encompasses several different mechanisms, including disease-related udder problems. Furthermore, this model can be used to study inter- and intra-individual variability in parameter values (e.g., by incorporating information on a single animal’s different lactations). Ultimately, this model could also be extended to other dairy livestock species.

This approach includes a variance model that helps ensure the validity of the overall model over the course of its development. By using maximum likelihood estimation, we obtained estimates that do not violate statistical assumptions. This work underscores the advantages of using a multidisciplinary approach to tackle a biological question, where a model is fit to observed data and a minimal number of biological mechanisms are represented. By using a biological model as a source for generating mathematical models, statistical models, and computer models, we can help ensure that biological reality is reflected. Therefore, the scientific knowledge and biological hypotheses included in the model ultimately inform the final interpretation of the results downstream. The model can thus identify gaps in knowledge that must be filled to better understand key biological mechanisms. In short, using this modelling approach, we were able to reduce a “rather complex” system to a “somewhat complicated” system that is coherent and understandable.

## Methods

### Modelling mean yield per milking session

We treated the udder as comprising a single quarter and a single compartment with an alveolar population of constant size for a given lactation period; the population is renewed only between lactations. Alveoli are epithelial cells that secrete and store milk. The population is split into three classes whose relative abundances changed over the lactation period. These classes are as follows: (1) non-activated alveoli, NC_I_(t); (2) activated alveoli (i.e., capable of producing milk), NC_A_(t); and (3) inactivated alveoli, NC_R_(t). Time is expressed in hours since t = 0, or the beginning of lactation, which generally precedes calving. Since the precise moment lactation begins is unknown, we estimated δ, or the length of time between the beginning of lactation and the first milking session. If alveolar population size, NC_T_, is constant over the lactation period, then, at any given moment, NC_I_(t) + NC_A_(t) + NC_R_(t) = NC_T_. We can therefore obtain the proportions of alveoli in the different classes at any point in time: N_I_(t) = NC_I_(t)/NC_T_; N_A_(t) = NC_A_(t)/NC_T_; and N_R_(t) = NC_R_(t)/NC_T_. When lactation begins, N_I_(0) = 1, N_A_(0) = 0, and N_R_(0) = 0. After lactation begins, and assuming alveolar activation and inactivation are random processes, changes in the relative proportions of the different alveolar classes can be described using a system of ordinary differential equations (SODE):

1 $$\{\begin{array}{c}\frac{d{N}_{I}(t)}{dt}=-{\lambda }_{A}\cdot {N}_{I}(t)\\ \frac{d{N}_{A}(t)}{dt}=+{\lambda }_{A}\cdot {N}_{I}(t)-{\lambda }_{R}\cdot {N}_{A}(t)\\ \frac{d{N}_{R}(t)}{dt}=+{\lambda }_{R}\cdot {N}_{A}(t)\end{array}$$

In this SODE, two parameters must be estimated: the rate of alveolar activation, λ_A_, and the rate of alveolar inactivation, λ_R_, which are considered to be constant over the lactation period.

The SODE has an explicit solution that is a function of the time since the beginning of lactation:

2 $$\{\begin{array}{c}{N}_{I}(t)=\exp (-\,{\lambda }_{A}\cdot t)\\ {N}_{A}(t)=({\lambda }_{A}/({\lambda }_{A}-{\lambda }_{R}))\cdot [\exp (-{\lambda }_{R}\cdot t)-\exp (-{\lambda }_{A}\cdot t)]\\ {N}_{R}(t)=1-{N}_{I}(t)-\,{N}_{A}(t)\end{array}$$

Milk secretion between successive milking sessions, denoted k and (k − 1), is a function of the time elapsed between the two, (t(k) − t(k − 1)); the proportion of activated alveoli during this interval, N_A_(t); the maximum volumetric capacity of the udder, V_max_; and the maximum secretion rate if all the alveoli are secreting milk, P_max_. The latter equals the product of per-alveolus secretion and the udder’s total number of alveoli, NC_T_, for a given lactation period. Milk secretion, Y_P_(t), is therefore defined by the following differential equation:

3 $$\frac{d{Y}_{P}(t)}{dt}={P}_{max}\cdot {N}_{A}(t)\cdot ({V}_{max}-\,{Y}_{P}(t)).$$

If the compartment, the site of milk secretion, is considered to be empty at time t(k − 1) after milking session (k − 1), this equation has an explicit solution:

4 $${Y}_{P}(k)=\,{V}_{max}\cdot [1-\exp (-{P}_{max}\cdot {\int }_{x=t(k-1)}^{x=t(k)}{N}_{A}(x)\cdot dx)]$$

When milking occurs twice daily, as in our study system, where the mean time between successive milking sessions was 14 (evening to morning) and 10 hours (morning to evening), the udder is far from reaching maximum capacity. Consequently, V_max_ and P_max_ cannot be estimated. Furthermore, because P_max_ is rather low, secretion can be approximated as follows:

5 $${Y}_{P}(k)=\,{V}_{max}\cdot {P}_{max}\cdot {\int }_{x=t(k-1)}^{x=t(k)}{N}_{A}(x)\cdot dx$$

In other words, when PL = V_max_ · P_max_, PL can be estimated, and then milk secretion at any given moment is a linear function of the proportion of activated alveoli:

6 $${Y}_{P}(k)=\,PL\cdot {\int }_{x=t(k-1)}^{x=t(k)}{N}_{A}(x)\cdot dx$$

This equation has an explicit solution and yields the following after integration:

7 $${Y}_{P}(k)=\,PL\cdot ({\lambda }_{A}/({\lambda }_{A}-{\lambda }_{R}))\cdot [{w}_{R}-\,{w}_{A}]\,$$

where

$${w}_{R}=\,(1/{\lambda }_{R})\cdot [\exp (-{\lambda }_{R}\cdot t(k-1))-\exp (-{\lambda }_{R}\cdot t(k))]$$

and

$${w}_{A}=\,(1/{\lambda }_{A})\cdot [\exp (-{\lambda }_{A}\cdot t(k-1))-\exp (-{\lambda }_{A}\cdot t(k))].$$

By definition, the parameter PL represents, to varying degrees, the udder’s total number of alveoli, udder maximum capacity, and the milk secretion rate of activated alveoli. Its value can therefore vary over the lactation period. More generally, certain factors could result in PL being constant within intervals but variable over the lactation period. Because we have no information *a priori* regarding these factors, the goal is to determine if they are present and account for them in the model as necessary. Using the residuals from an initial model in which PL was assumed to be constant over the lactation period, we could choose the intervals to be included in the final model.

If we assume that the udder (i.e., cisterns) has not been completely emptied at the end of each milking session, then it clearly becomes important to consider the milk retention rate, or π, which is assumed to be constant over the lactation period. For a given milking session, k, the milk extracted, Y_E_(k), is thus the sum of the milk produced since the previous session, Y_P_(k), to which is added the residual milk (carryover) from the previous session Y_R_(k − 1) and from which is subtracted the residual milk from the current session, Y_R_(k). Therefore,

8 $${Y}_{E}(k)\,=\,{Y}_{P}(k)-{Y}_{R}(k)\,+\,{Y}_{R}(k-1){\rm{.}}$$

If we consider that

9 $${Y}_{R}(k)\,=\,\pi \cdot ({Y}_{P}(k)+{Y}_{R}(k-1)),$$

then

10 $${Y}_{E}(k)\,=\,(1-\pi )\cdot ({Y}_{P}(k)+{Y}_{R}(k-1)).$$

Here, we are thus assuming that the probability of milk extraction during a given session is (1 − π). Using the residuals from an initial model in which this parameter was held constant over the lactation period, we could identify any extreme retention rate values during milking sessions. Including retention rate in the model allows us to account for the autocorrelation commonly observed in milk yield over successive sessions.

Using a model in which milk retention rate, π, and milk production rate, PL, are constant over the lactation period, we can exploit the normalized estimated residuals to characterize the following sources of variance:

1. Variance in the milk retention rate that is specific to a given milking session, which reflects the physiological reality that retention rates can be extreme for certain sessions. Extreme values were considered to occur when the standardized residual had a probability of less than 0.001 of occurring. These cases were estimated in the final model by considering rate values that were “too” different from the mean rate.
2. Variance over time in the secretion rate, which reflects the physiological fact that the values of parameters associated with milk secretion change over time. Such variance can be identified by analysing changes in the mean and variance of the model’s standardized residuals over the course of successive milking sessions. This task was accomplished using the cpt.meanvar function in the R package changepoint^76^, with which probable parameter change points can be statistically identified (alpha level of 0.05). We thus obtained the parameter PL(k), which is constant within intervals over the lactation period. Since PL_max_ = max(PL(k)), we could introduce the parameter β(k), which is equal to PL(k)/PL_max_ and varies between 0 and 1. It therefore characterises the udder’s milk production potential over the lactation period. However, it is somewhat more difficult to interpret β(k) because, by definition, it represents the effects of several different physiological mechanisms. For instance, its value is influenced by the secretion rate, which can vary over the lactation period based on an animal’s diet, by udder swelling when an animal is put out to graze, by the proportion of activated alveoli, and by milk “build-up” when the alveolar compartment contracts as milking begins.

By accounting for these two types of variation in the model, we obtained independent, standardized residuals that follow a normal distribution, making it possible to validate the adjusted model. From a methodological standpoint, the two categories of variation correspond to conditional elements, which are factors that introduce variation over the lactation period and for which precise information is rarely available beforehand. Modelling such variance may therefore be helpful in identifying such factors *a priori* and thus making it possible to test their effects *a posteriori*.

Using estimated milking-session means and variances (obtained exclusively when residuals are uncorrelated), we could estimate daily means and variances for milk yield, by summing the results for a day’s morning and evening sessions. We then validated this daily-yield model using estimated normalised residuals from the milking-session models.

Wood’s model, which is an empirical model that describes the daily lactation curve of a dairy cow, is the standard reference model. It employs a gamma function that mirrors milk yield over the course of a day *x*_*j*_:

11 $${Y}_{Ej}=a\cdot {x}_{j}^{b}\cdot \exp (-c\cdot {x}_{j})+{\varepsilon }_{j}.$$

However, its three parameters (a, b, and c) do not have a clear biological interpretation and the parameters are estimated using least squares.

### Modelling variance in yield per milking session

To model session-specific variance in yield, we broke down total variance into its different components. For the variance associated with measurement error, we considered that, for each milking session k, the amount of milk measured, or Y_M_(k), is a random variable that follows a log-normal distribution, whose level of precision is determined beforehand using a coefficient of variation, CV_M_, equal to 0.025 (or 2.5%). The model describing measured milk yield is as follows: *Y*_*M*_(*k*) = *Y*_*E*_(*k*) · *exp*(*ε*), where *E*(*ε*) = 0 and *Var*(*ε*) = *CV*_*M*_^2^. The mean is calculated using *E*(*Y*_*M*_(*k*)) = *E*(*Y*_*E*_(*k*)), since when |ε| < 1, $E(exp(\varepsilon ))=exp(E(\varepsilon ))=1.$ To find the variance, we use

12 $$Var({Y}_{M}(k))=\,E{({Y}_{E}(k))}^{2}\cdot Var(\varepsilon )+Var({Y}_{E}(k)),$$

where Y_E_(k) is also a random variable.

For the variance associated with the amount of milk extracted, Y_E_(k), we consider that, for each milking session k, Y_E_(k) is a random variable whose mean and variance are estimated using

13 $$E({Y}_{E}(k))=\,(1-\pi )\cdot (E({Y}_{P}(k))+E({Y}_{R}(k-1)))$$

and

14 $$Var({Y}_{E}(k))=(E({Y}_{P}(k))+E({Y}_{R}(k-1)))\cdot \pi \cdot (1-\pi )+{(1-\pi )}^{2}\cdot Var({Y}_{P}(k))+{(1-\pi )}^{2}\cdot Var({Y}_{R}(k-1)),$$

respectively.

The milk produced since the previous milking session, Y_P_(k), is also a random variable. Calculating the variance in Y_E_(k) requires an iterative approach involving the variance in the amount of residual milk:

15 $$Var({Y}_{R}(k))=(E({Y}_{P}(k))+E({Y}_{R}(k-1)))\cdot \pi \cdot (1-\pi )+{\pi }^{2}\cdot Var({Y}_{P}(k))+\,{\pi }^{2}\cdot Var({Y}_{R}(k-1)),$$

where E(Y_R_(0)) = 0 and Var(Y_R_(0)) = 0.

Variance in milk secretion within intervals, Y_P_(k), can be due to either random variability in β(k) or random variability in the mean proportion of activated alveoli, MN_A_(k), for the current interval. Therefore, the mean can be found as follows:

16 $$E({Y}_{P}(k))=\,P{L}_{max}\cdot E({\rm{\beta }}({\rm{k}}))\cdot (t(k)-t(k-1))\cdot E(M{N}_{A}(k))$$

where

$$E(M{N}_{A}(k))=\,\frac{{\int }_{x=t(k-1)}^{x=t(k)}{N}_{A}(x)\cdot dx}{(t(k)-t(k-1))}.$$

Variance is equal to

17 $$Var({Y}_{P}(k))=\,{V}_{PL}(k)+{V}_{PA}(k),$$

where

18 $${V}_{PL}(k)=P{L}_{max}\cdot (t(k)-t(k-1))\cdot E(M{N}_{A}(k))\cdot E(\beta (k))\cdot (1-E(\beta (k)))$$

and

19 $${V}_{PA}(k)={[P{L}_{max}\cdot E(\beta (k))]}^{2}\cdot (t(k)-t(k-1))\cdot \varphi \cdot E(M{N}_{A}(k))\cdot (1-E(M{N}_{A}(k))).$$

The latter equation includes a parameter, *ϕ*, that must be estimated. It is a dispersion parameter that can handle underdispersion and thus account for interdependence among alveoli.

For a given milking session, total variance *Var*(*Y*_*M*_(*k*)) can be broken down into variance due to measurement error,

20 $${V}_{mes}(k)=E{({Y}_{E}(k))}^{2}\cdot Var({\epsilon });$$

variance due to milk retention,

21 $${V}_{ret}(k)=[E({Y}_{P}(k))+E({Y}_{R}(k-1))]\cdot \pi \cdot (1-\pi );$$

milk carryover,

22 $${V}_{rec}(k)={\pi }^{2}\cdot Var({Y}_{R}(k-1));$$

the proportion of PL_max_,

23 $${V}_{pro}(k)={(1-\pi )}^{2}\cdot {V}_{PL}(k);$$

and the proportion of activated alveoli,

24 $${V}_{alv}(k)={(1-\pi )}^{2}\cdot {V}_{PA}(k).$$

Therefore, for each milking session, we obtained:

25 $$Var({Y}_{M}(k))={V}_{mes}(k)+{V}_{ret}(k)+{V}_{rec}(k)+{V}_{pro}(k)+{V}_{alv}(k).$$

We quantify the relative contribution of each variance component by dividing the component’s value by total variance, for each milking session and for the full lactation period. To determine the amount of variance explained by the model, we find the ratio of the total variance as calculated above to the total residual variance remaining after maximum likelihood estimation.

We also modelled the variance associated with interval duration by treating the latter as a random variable for which only the mean and variance are known. The relevant equations are as follows:

26 $${V}_{tim}(k)={(1-\pi )}^{2}\cdot {[P{L}_{max}\cdot E(\beta (k))]}^{2}\cdot {[\frac{{\lambda }_{A}}{{\lambda }_{A}-{\lambda }_{R}}]}^{2}\cdot {[d{w}_{R}-d{w}_{A}]}^{2}\cdot Va{r}_{T},$$

where

$$d{w}_{R}=\exp (-{\lambda }_{R}\cdot t(k-1))\cdot \exp (-{\lambda }_{R}\cdot {\rm{\bigtriangleup }}t(k)),$$

$$d{w}_{A}=\exp (-{\lambda }_{A}\cdot t(k-1))\cdot \exp (-{\lambda }_{A}\cdot {\rm{\bigtriangleup }}t(k))$$

Δt(k) = 14.0 if k is a morning session, Δt(k) = 10.0 if k is an evening session and *Var*_*T*_ = *0.25*.

For a given milking session k, total variance is therefore:

27 $$Var({Y}_{M}(k))={V}_{mes}(k)+{V}_{ret}(k)+{V}_{rec}(k)+{V}_{pro}(k)+{V}_{alv}(k)+{V}_{tim}(k).$$

### Applying and validating the model

Model parameter estimation was carried out using the maximum likelihood estimator obtained from the milking-session model. Consequently, if Y_O_(k) is observed milk yield for milking session k, then the model’s standardised residuals, (*Y*_*O*_(*k*) − *E*(*Y*_*M*_(*k*)))/*Var*(*Y*_*M*_(*k*))^*1/2*^, follow a normal distribution if the model is accurate. The model parameters were therefore estimated using the maximum likelihood estimator, by integrating the mean and variance models. To ensure the different parameters had valid values, we used the appropriate transformations. For instance, when a parameter ϴ can only have positive values, we used the transformation ϴ = exp(α), where the domain of α is all real numbers. When a parameter ϴ is only meaningful at values between 0 and 1, we used the transformation ϴ = exp(−exp(α)), where the domain of α is once again all real numbers.

The nlm function in R was used to minimize the model’s maximum likelihood function (see Supplementary Note: “Parameter estimation using the nlm function in R.” online). We obtained the estimator variances along the main diagonal by inversing the Hessian matrix. The parameters’ variances and 95% confidence intervals were then calculated after inversely transforming their estimates and their bounds, by exploiting the normality of the maximum likelihood estimators.

We then validated the milking-session and daily-yield models using an analysis of the model’s standardized residuals, which were normally distributed and uncorrelated, meeting statistical assumptions. Normality was tested using a Kolmogorov-Smirnov (KS) test. A Box-Pierce^77^ test was used to confirm that the residuals were not auto-correlated. The results were visualised by plotting the empirical cumulative distribution function (R function ecdf) and estimates of the partial autocorrelation function (R function pacf), respectively.

We validated the models for session-specific means and variances by simulating different sets of milking-session observations while accounting for the different conditional elements included in the variance model. We obtained a large number of simulated datasets (n = 1,000), which allowed us to estimate session-specific means and total variances. Strong correlations between the estimates obtained from the models and the simulations for the means, total variances, and 95% confidence intervals helped validate the model structure used to arrive at the values of those statistics. Taken together, the simulated datasets obtained for the full lactation period allowed us to confirm the robustness of the milking-session and daily-yield models (see Supplementary Figs 2 and 3).

We determined the variance due to the parameter estimators by integrating the estimator variance-covariance matrix into the simulations. Over the lactation period, the variance associated with estimator precision represented 4% of the total estimated variance. We found that, for the first milking sessions, the greatest influence was wielded by the parameter estimating the duration δ between the beginning of lactation and the first milking session.

A pilot simulation program was developed to help visualise the influence of the milk-yield model’s four main parameters — *λ*_*A*_, *λ*_*R*_, *PL*_*max*_, and *π* — on the mean and variance models and on the principal criteria for model validation. It uses the R packages tcltk and tkrplot (see Supplementary Fig. 6).

### Data Availability

The data are available in a ZIP file (4.28 MB), “**ModelPL-SR-PG-20170425**”, which is located in an INRA repository at the permanent web address: “http://epia.clermont.inra.fr/plsrpg”. In exchange for access to the file, we simply request that the interested party provide his or her email. The following information is included in the ZIP file. (1) The lactation data that was analysed in this manuscript and lactation data for two other dairy cattle species—the Holstein and the Montbéliarde. They are located in the “**Data**” directory; data on different lactation ranks (two to six) are also included. This information can be used to illustrate how the model can be generalised. (2) The numerical results and graphs in pdf format are located in the “**GraphPDF**” directory. (3) The R scripts we used are in the “**ProgR**” directory. They include the scripts for model parameter estimation, the simulation integrating random parameter variability, the automatic output of numerical results and graphs in pdf format, and the running of the pilot simulation. (4) In the latter directory, there is also a general script, “**ScriptRgeneral.txt**”, for implementing the other scripts in a logical order. We chose to leave all the scripts in R so that they are easy to read and evaluate if one has a basic understanding of the R language. All of the functions described above will ultimately be grouped into an R package once they have been optimized and once estimation procedures have been written in C. An example library for the Windows OS is included in “**ProgC**” directory.

## Electronic supplementary material

Supplementary Information
