# Supplementary material for: A new standard model for milk yield in dairy cows based on udder physiology at the milking-session level
Source: Sci Rep. 2017 Aug 21;7:8897. doi: 10.1038/s41598-017-09322-x (PMC5567198; doi:10.1038/s41598-017-09322-x)
Supplement: Supplementary file 1 — Supplementary Information [file 41598_2017_9322_MOESM1_ESM.pdf]

A new standard model for milk yield in dairy cows based on udder physiology at the milking-session level

Patrick Gasqui* & Jean-Marie Trommenschlager

Supplementary Information

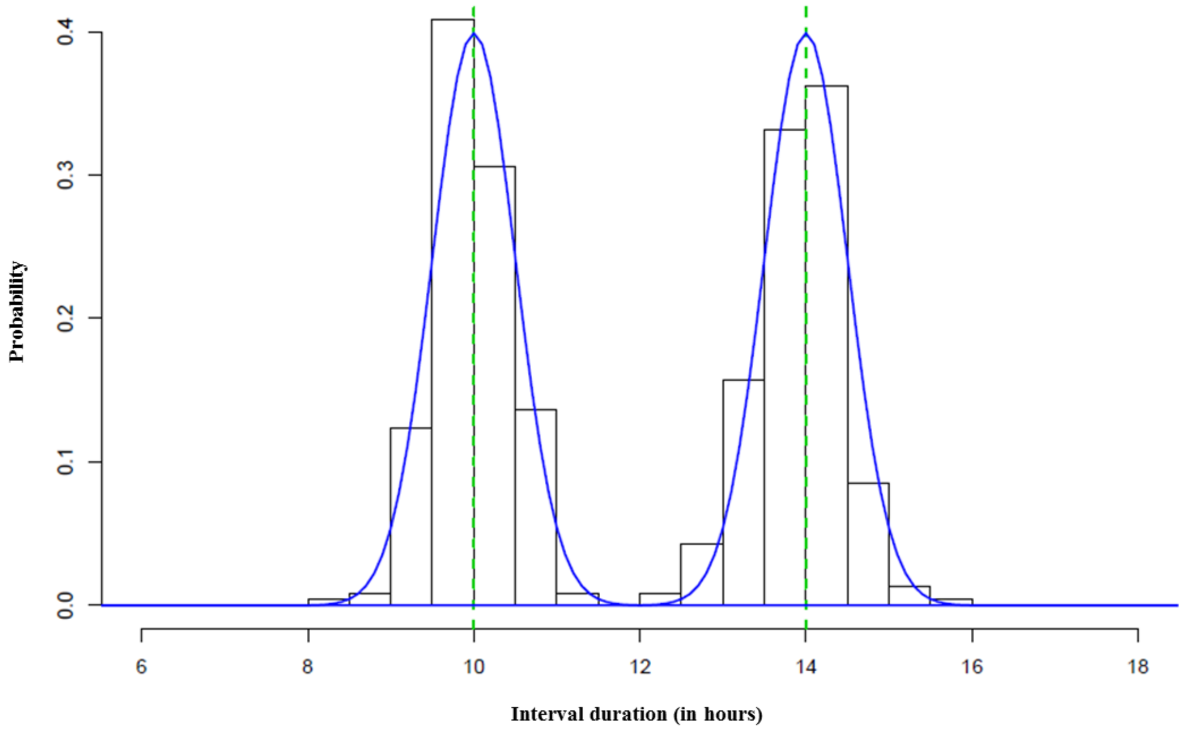

Supplementary Figure 1. Distribution of between-session duration. Histogram of interval duration (black bars), with a mean of 10 and 14 hours for the morning and evening milking sessions, respectively (dashed green lines). The adjusted distribution of interval duration (solid blue curve) follows a normal distribution, and the morning and evening milking sessions have the same standard deviation of 0.50.

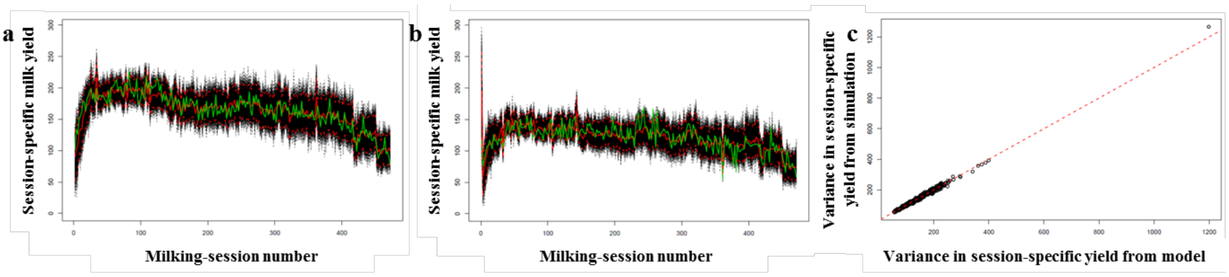

Supplementary Figure 2. Session-specific milk yield from the simulation and the model. a,b, simulated yield (n=1,000; black line), observed yield (green line), and model-estimated yield±95% CI (solid and dashed red lines, respectively) for the morning (**a**) and evening (**b**) milking sessions. **c**, plot of the total session-specific variance in milk yield found using the model versus the simulation (dashed red best-fit line).

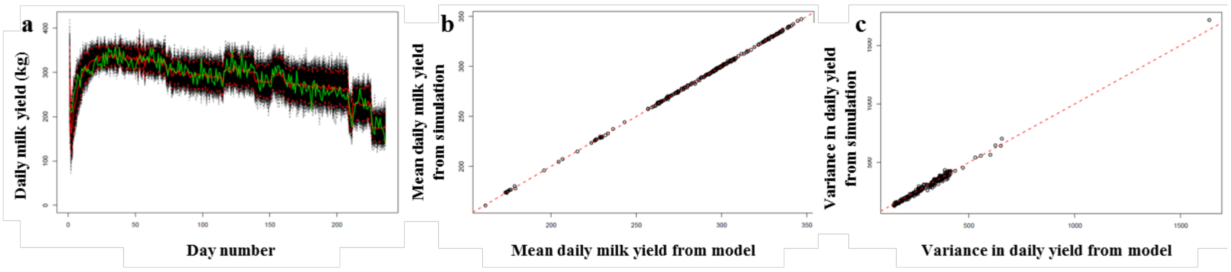

Supplementary Figure 3. Daily milk yield from the simulation and the model. a, simulated yield [n=1000] (black line), observed yield (green line), and model-estimated yield±95 CI (solid and dashed red lines, respectively). **b**, plot of mean daily milk yield found using the model versus the simulation (dashed red best-fit line). **c**, plot of the total daily variance in milk yield found using the model versus the simulation (dashed red best-fit line).

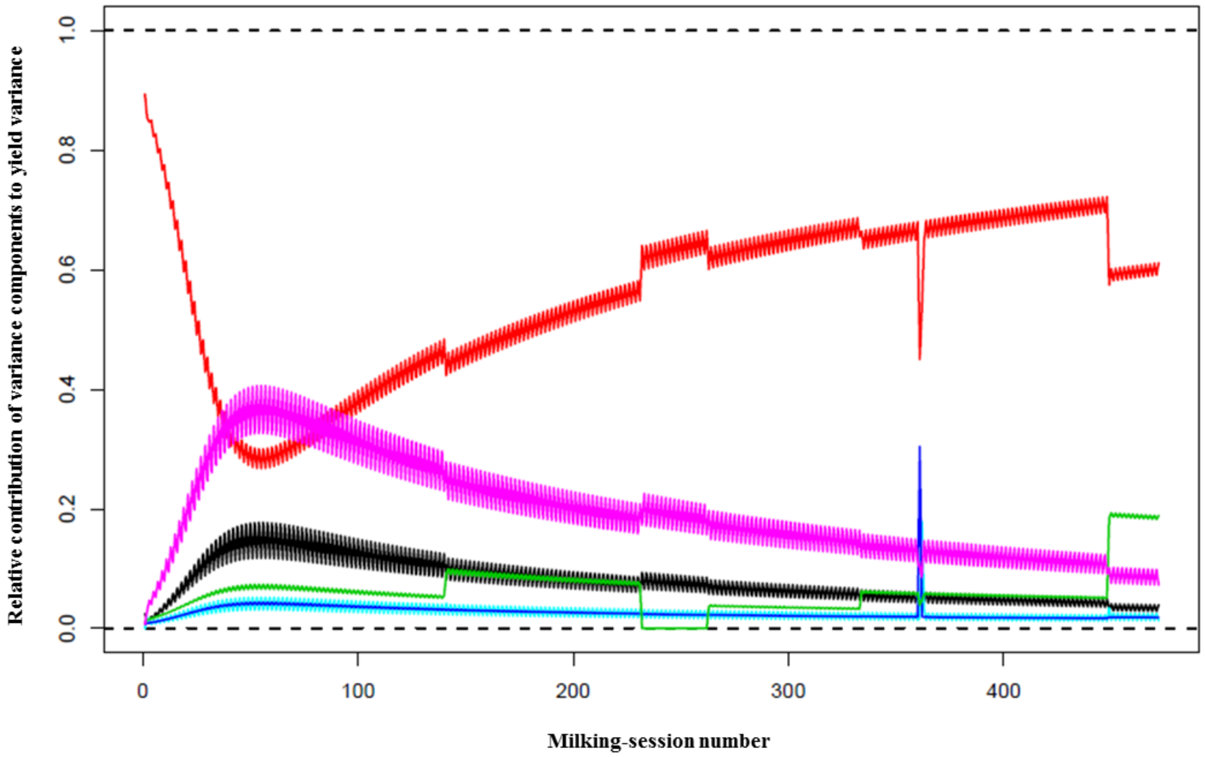

Supplementary Figure 4. Relative contribution of variance components to total variance over the lactation period. Session-specific estimates (% provided is the estimate for whole lactation period): proportion of activated alveoli (62.1%; red line), interval duration (18.8%; magenta line), measurement error (7.9%; black line), β (6.1%; green line), π (2.6%; blue line); and milk carryover (2.5%; cyan line).

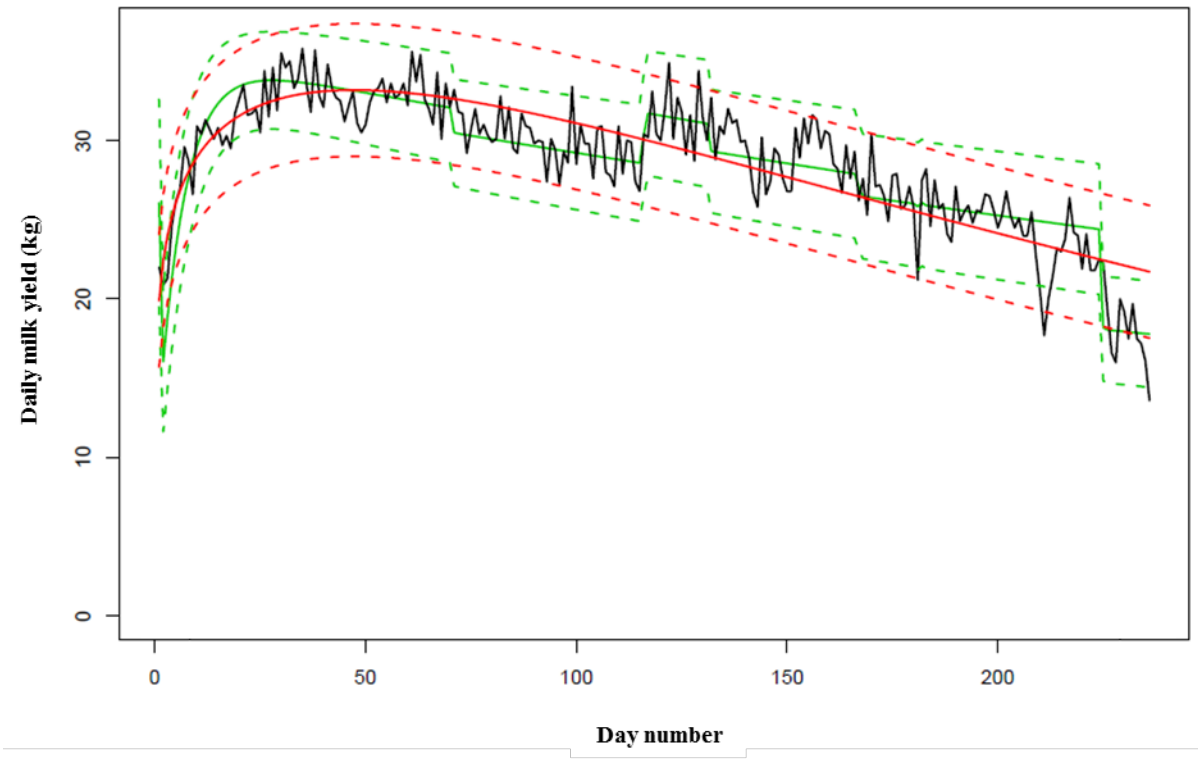

Supplementary Figure 5. Observed versus estimated milk yield full days when precise interval duration was unknown. Observed yield is represented by a solid black line. Model-estimated yield and the 95% CI are represented by green lines (solid and dashed, respectively). The estimated yield from Wood's model and the 95% CI are represented by red lines (solid and dashed, respectively).

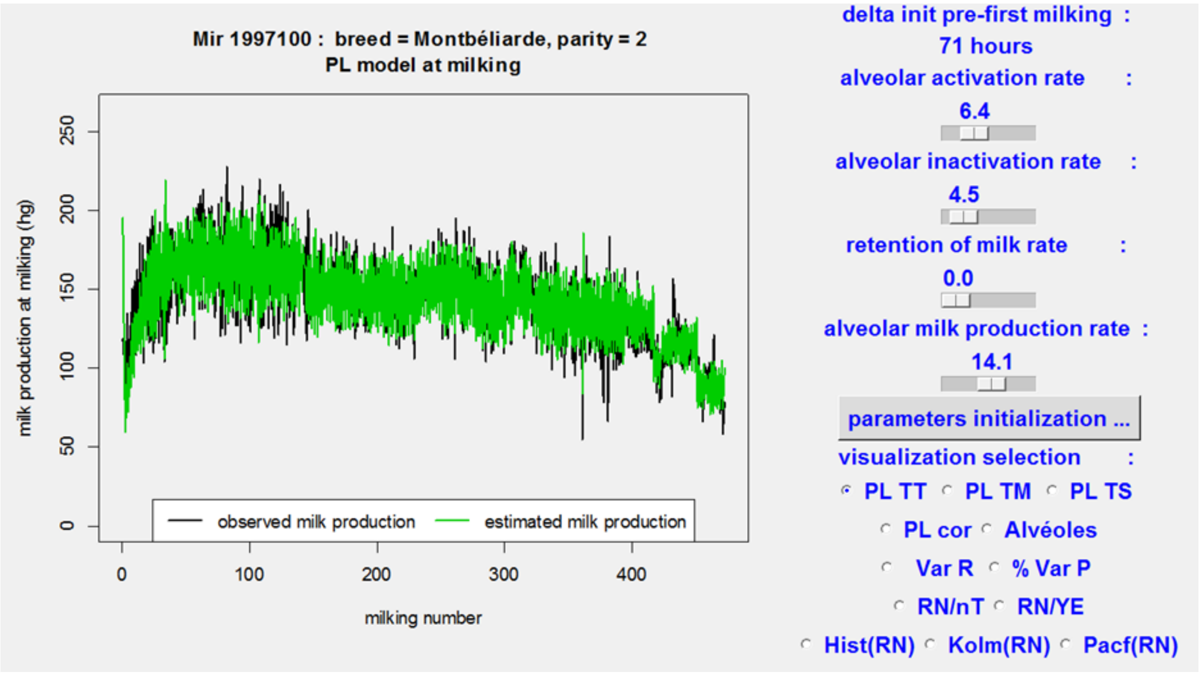

Supplementary Figure 6. Image illustrating the simulation used to estimate parameter influence in the milk-yield model.

Note: “Parameter estimation using the nlm function in R.”

The R function nlm carries out the non-linear minimisation of target functions, and we used it to estimate all the model parameters at once. Based on the output, we can see that this process seemed to run smoothly.

```
resNLM <- nlm( fctMVMPLTempsEV, pinitEVG, xDSTemps, hessian = TRUE, iterlim = 400 )
resNLM
```

```
$minimum
[1] 1841.758

$estimate
 [1]  4.26236099  -5.04385102 -10.00942585  1.16457498  2.64294427 -0.61988279  0.24482785 -0.06540155 -0.31801924
[10] -0.19003679 -0.44434607

$gradient
 [1] -1.947076e-06 -1.001326e-05 1.076339e-05 -1.922155e-06 -1.184601e-04 -3.092282e-07 -3.722107e-06 -2.923002e-05 1.542617e-05
[10]  2.650040e-06 -1.026251e-05

$hessian
            [,1]         [,2]          [,3]       [,4]         [,5]        [,6]          [,7]          [,8]          [,9]
 [1,] 128.8393767  162.9949969   17.7052440   6.021159   227.176031   0.5471389 -7.323512e-01 -8.099211e+01 -1.317717e+00
 [2,] 162.9949969  431.9892014    0.2630941  16.467608  1704.429236  -1.9908612 -5.430297e+00 -1.779131e+02 -2.898310e+00
 [3,]  17.7052440    0.2630941 1053.4040257 -13.788833 -6972.099545  83.9945642 -2.007414e+00 -3.518729e+03 -9.157020e+01
 [4,]   6.0211593   16.4676082  -13.7888326 141.939706    10.179624 -21.6276137 -2.195712e+01 -7.084863e+01 -9.908871e+00
 [5,] 227.1760312 1704.4292363 -6972.0995449  10.179624 79039.766408 356.1417795  1.499545e+00  2.702644e+04  4.272002e+02
 [6,]   0.5471389   -1.9908612   83.9945642 -21.627614   356.141779 130.1878228 -7.449898e-01  7.913040e+01  2.805268e+00
 [7,]  -0.7323512   -5.4302973   -2.0074140 -21.957123     1.499545  -0.7449898  5.453351e+01 -5.385095e+00 -2.273737e-05
 [8,] -80.9921150 -177.9130798 -3518.7290905 -70.848629 27026.440605  79.1304046 -5.385095e+00  2.677550e+04  6.264531e+00
 [9,]  -1.3177168   -2.8983095  -91.5702003  -9.908871   427.200218   2.8052682 -2.273737e-05  6.264531e+00  4.174649e+02
[10,]  -6.5098953  -14.3184707 -470.6328809   5.088215  2112.712788  15.4587042 -2.273737e-05 -2.273737e-05  1.625813e+00
[11,]  -4.0749252   -8.9627747 -312.2035878  -6.079477  1325.320906  10.1367732 -2.273737e-05 -2.273737e-05  0.000000e+00
              [,10]         [,11]
 [1,] -6.509895e+00 -4.074925e+00
 [2,] -1.431847e+01 -8.962775e+00
 [3,] -4.706329e+02 -3.122036e+02
 [4,]  5.088215e+00 -6.079477e+00
 [5,]  2.112713e+03  1.325321e+03
 [6,]  1.545870e+01  1.013677e+01
 [7,] -2.273737e-05 -2.273737e-05
 [8,] -2.273737e-05 -2.273737e-05
 [9,]  1.625813e+00  0.000000e+00
[10,]  2.096931e+03  4.776712e+00
[11,]  4.776712e+00  1.314261e+03

$code
[1] 1

$iterations
[1] 78
```

1 – The output code was 1, which, according to the nlm documentation, is the best value you can obtain.

```
$code
[1] 1
```

"nlm R documentation"
"code : an integer indicating why the optimization process terminated

- **1 : relative gradient is close to zero, current iterate is probably solution.**
- 2 : successive iterates within tolerance, current iterate is probably solution.
- 3 : last global step failed to locate a point lower than estimate. Either estimate is an approximate local minimum of the function or steptol is too small.
- 4 : iteration limit exceeded.
- 5 : maximum step size stepmax exceeded five consecutive times. Either the function is unbounded below, becomes asymptotic to a finite value from above in some direction or stepmax is too small."

2 – The number of iterations was 78, which is not very high, given that we set the maximum number of iterations to 400 (via interlim); the default value is 100.

```
$iterations
[1] 78
```

It is perhaps worth noting that nlm uses a Gauss-Newton-type algorithm, which is not necessarily optimal in complex non-linear situations. On the one hand, we would have preferred a Gauss-Marquart-type algorithm, which is more optimised for cases such as ours and more rapidly converges towards a solution (i.e., requires far fewer iterations). However, no such option exists for nlm. On the other hand, we did want to use a general non-linear mimisation function such as nlm to demonstrate that parameter estimation can be efficacious and robust when commonly available procedures are used.

3 – In nlm, numerical derivatives are used for the gradient vector and the Hessian matrix. Algorithm speed increases when the vector and the matrix are explicitly calculated.
The process yields a Hessian matrix whose inverse is easy to obtain using the solve function. This shows that all the parameters could be estimated. From the Hessian matrix, we obtained a variance-covariance matrix for all of the parameters; the main diagonal was the linear vector of the variances.

```
matHes   <- resNLM$hessian
matVCW  <- solve(matHes)
varParEL <- diag(matVCW)
```

The fact that we obtained reasonable and coherent values for these variance estimators is another sign that parameter estimation ran smoothly. We can thus feel confident that all of the parameters could be estimated.

4 – To show that the values presented in the final versions of the tables (1, 2, 3, and 4) correspond to what was obtained using the nlm function, we present the output (resNLM – see above).

```
espParB <- c(exp(resNLM$estimate[1:3]), exp(resNLM$estimate[6]))
> espParB
[1] 7.097736e+01  6.448866e-03  4.497401e-05  5.380075e-01
```

Delta time δ (hour) = 70.98

Activation rate λ_A (per hour) = 64.10^{-4}

Inactivation rate λ_R (per hour) = 45.10^{-6}

Inflation variance factor ϕ = 0.54

```
espParT <- c(exp(-exp(resNLM$estimate[4])) ,exp(-exp(resNLM$estimate[c(7:(7+nbTV-1))])))
> espParT
[1] 0.04057673  0.27876075
```

Retention rate $\pi(k)$ “for 33 and 361” = 0.28

Retention rate $\pi(k)$ “for others” = $\min(\pi(k))$ = 0.04

```
espParP <- c(exp(resNLM$estimate[5]) ,
exp(resNLM$estimate[5])*exp(resNLM$estimate[(7+nbTV):length(resNLM$estimate)]) )
> espParP
[1] 14.054523  13.164749  10.225913  11.622089  9.012371
```

Secretion rate PL(k) estimate (in hg per hour) of table 4 = 14.1 13.2 11.6 10.2 9.0

Secretion rate max (in hg per hour) = max(PL(k)) = 14.1
